# Reciprocity as a Foundation of Financial Economics

**DOI:** 10.1007/s10551-014-2257-x

**Published:** 2014-07-06

**Authors:** Timothy C. Johnson

**Affiliations:** Maxwell Institute for Mathematical Sciences and Department of Actuarial Mathematics and Statistics, Heriot-Watt University, Edinburgh, EH14 4AS UK

**Keywords:** Financial economics, Reciprocity, Communicative action, Pragmatism

## Abstract

This paper argues that the subsistence of the fundamental theorem of contemporary financial mathematics is the ethical concept ‘reciprocity’. The argument is based on identifying an equivalence between the contemporary, and ostensibly ‘value neutral’, Fundamental Theory of Asset Pricing with theories of mathematical probability that emerged in the seventeenth century in the context of the ethical assessment of commercial contracts in a framework of Aristotelian ethics. This observation, the main claim of the paper, is justified on the basis of results from the Ultimatum Game and is analysed within a framework of Pragmatic philosophy. The analysis leads to the explanatory hypothesis that markets are centres of communicative action with reciprocity as a rule of discourse. The purpose of the paper is to reorientate financial economics to emphasise the objectives of cooperation and social cohesion and to this end, we offer specific policy advice.

## Introduction

Paul Rubin has coined the term ‘emporiophobia’, meaning the fear of markets (Rubin 2014). Rubin argues that, not only is emporiophobia widespread, it is manifested in legislation that has economic implications detrimental to society’s well being. He identifies the source of emporiophobia as being in an over-emphasis in economics of the ‘competition’ metaphor at the expense of the ‘cooperation’ metaphor and the antidote to emporiophobia is for economists to switch this focus in their arguments. This paper supports Rubin’s argument and we will present a stronger case that reciprocity, a basis of cooperation, is at the heart of financial economics. We make the stronger case to enable our beliefs to impact people’s experiences of markets.

Our case starts by arguing that the foundational theory of financial mathematics, the Fundamental Theorem of Asset Pricing (hereafter ‘FTAP’), has its basis in the Aristotelian virtue ‘Justice’. The FTAP is the theory underpinning modelling frameworks such as Black–Scholes–Merton, Cox–Ross–Rubinstein, Heath–Jarrow–Morton and the LIBOR Market Models and it is the central theory of contemporary mathematical approaches to pricing derivatives employed in financial economics. Its significance is in unifying various strands in financial economics: Samuelson and Merton’s use of stochastic calculus; CAPM, developed by Treynor and Sharpe; martingales, employed by Fama in the development of the Efficient Markets Hypothesis; Arrow and Debreu’s concept of incomplete markets. In accomplishing this unification, it represents a paradigm for financial economics.

The argument is made by synthesising an understanding of contemporary financial mathematics with historical scholarship. Our argument is based on Aristotelian ethics, where a correspondence between Justice in exchange, reciprocity and fairness, and a relationship to mathematics is identified in Book V, Chapter 5 of *Nicomachean Ethics* (Broadie and Rowe 2011, pp. 1132b21–1134a16; Judson 1997). We describe how the concept of probability emerges in the thirteenth century and develops into a mathematical theory of probability in the ethical examination of commercial practice. We then present the main claim, starting with a review of the FTAP we then provide the core argument that explains the equivalence between contemporary and late seventeenth century ideas. We follow this analysis by offering an explanation as to why the relationship between financial mathematics and commercial morality became obscured in the nineteenth century.

The moral basis of financial economics has been addressed by a variety of authors, for example, Jackson (2010) tackles it tangentially by addressing failures in the curricula of Business Schools, an issue examined in detail by West (2012). Pre-dating the events of 2007, Horrigan (1987) undertakes a critical analysis of the moral consequences of certain theories of financial economics that he labels ‘the New Finance’, including the Efficient Markets Hypothesis, CAPM and options pricing models. His conclusion is that because the financial world is objectified it becomes “not a nice place ethically”. Frankfurter and McGoun (Frankfurter et al. 2002; Frankfurter 2006) argue that there is a fundamental problem with “The methodological foundation of the established finance paradigm, which for simplicity I will call the EMH” (Frankfurter 2006, p. 134) and offer an alternative paradigm. There is significant overlap between our position and Frankfurter (2006) but with a significant difference; we argue that the EMH is intrinsically fair and our objective is to make this explicit.

Both Horrigan and Frankfurter and McGoun highlight the issue that there is a dichotomy between fact and value that originates in Hume and even authors who reject an analytic/synthetic distinction in philosophy, such as Quine, do not admit that there can be a moral dimension to mathematics (Misak 2002, p. 85). This fact/value dichotomy creates a barrier that inhibits ethical analysis of overtly mathematical themes, explaining the paucity of literature addressing the ethical implications of financial economics, as compared to scholarship on ethics in other technology based professions. In our analysis, we ignore the fact/value dichotomy by adopting an approach founded on Pragmatic philosophy (Putnam 2002). Pragmatism is especially relevant to finance because it addresses the thorny issue of truth when we cannot rely on objectivity, neutrality and determinism and because it acknowledges the role of ethics in science. Specifically, by rejecting the ideology of the fact/value dichotomy, we claim that the principle heuristic for the technical results of the FTAP, the ‘Dutch book argument’, is equivalent to the ‘Golden Rule’—“Do unto others as you would have them do unto you”. The consequence of this claim is that the principle of ‘no arbitrage’ in pricing contingent claims, at the heart of ‘New Finance’, is infused with the moral concept of reciprocity, or fairness, which is integral in cooperation.

The argument is centred on financial markets, but the identification of the basis of asset pricing in reciprocity has broader economic significance. For example, consider Pindyck (2013) that argues that economic cases for taking actions today to mitigate the long term consequences of climate change rest on taking a very low discount rate, and so are difficult to justify with mainstream financial theory. Low rates are justified by rejecting profit seeking market rates in favour of the principle of inter-generational reciprocity and it is difficult, as demonstrated in Stern (2008, II.B) to do this persuasively using conventional economic arguments. The case that finance *is* based on reciprocity, not on profit maximisation, immediately justifies the arguments in Stern for inter-generational reciprocity, in particular, and long-term investment at low, but sure, returns, in general.

The argument that contemporary asset pricing is infused with the moral concept of Justice that we present can be used: to challenge beliefs concerning the immorality of markets, highlighted by Rubin; to present the ‘New Finance’ as having ethical foundations, redressing Horrigan’s concerns; and to support Stern’s principle of inter-generational reciprocity in investment analysis. However, in order to achieve our objective of contributing to a re-orientation of finance such that it focuses on the objective social cohesion we need to robustly justify our claim. To motivate this justification we will identify some issues raised in Rubin’s speech.

Rubin concludes his argument with the following remark

[The market] system is moral because it maximises human welfare. It provides the most goods and services feasible, and provides them in the least cost way. The lives of ordinary people under capitalism are as happy as it is possible for them to be. No other system can make this claim. This measure of morality is a pure output based measure: *capitalism is moral because of what it produces*. People do not fully grasp the moral benefits of capitalism because we tend to focus on competition, which is only a tool, rather than on cooperation, which is the actual goal of the economic system. (Rubin 2014, my italics)

In light of persistent crises in finance since 2007 many argue, reasonably and rationally, that ‘capitalism is *immoral* because of what it produces’. Both the US and UK legislatures challenge the morality of contemporary markets. The Financial Crisis Inquiry Commission (FCIC) concluded that in the lead up to The Crisis there had been a “systemic breakdown in accountability and ethics” (FCIC 2011). The Parliamentary Commission on Banking Standards (PCBS 2013) pointedly titled their comprehensive report “Changing Banking for Good”, emphasising that finance should reorientate itself in an explicitly moral direction.

Rubin’s suggestion that economists should emphasise cooperation in their intra-disciplinary discussions will not be sufficient to redirect finance in the time-frame society demands. The problem Rubin faces is the one that Cheryl Misak addresses when she asks “Why must we value cooperation and equality” (Misak 2002, p. 26). Simply stating that cooperation is a preferable metaphor will not change the attitudes of a trader who believes manipulation is justified in the quest for profits. Rubin’s closing remark, apart from the final sentence, do not challenge the trader’s beliefs. This observation entails that we focus on Rubin’s final sentence and the actual goal of the financial system. To this end we shall adopt the Aristotelian position that profit is a good external to financial markets, the good internal to the markets is the transfer of commodities, and credit, in support of social cohesion. This observation is in the spirit of MacIntyre (2013, Chap. 14, esp. p. 188) and, with reference to Rubin’s discussion of the use of sporting metaphors in economics, it invites the comment that the good internal to sport could well be the development of teamwork or physical excellence, not the objective of winning.

Another issue that emerges out of Rubin’s argument is more clearly highlighted in Caplan’s earlier identification of emporiophobia, as an anti-market bias, in his critique of democracy (Caplan 2007). Caplan’s argument is essentially that democracies fail because the voting public is unable to rationally identify what is good for them, such as the profit-seeking market mechanism in distributing resources. There are a number of problems with Caplan’s thesis. The experience from the natural and physical sciences is that the public cannot be brought to appreciate or correctly interpret scientific results just through better education in science; public understanding *of* science has been superseded by public engagement *with* science. The relevance of this observation is that while there have been two significant environmental disasters since 2009—Deep Water Horizon (2010) and Fukishima Daiichi (2011)—which appear to have been resolved in public opinion, financial disasters have not. The implication is that intra-disciplinary discussions are not going to resolve the issue of emporiophobia.

A second problem is that Rubin highlights the impact of emporiophobic legislation while Caplan’s argument has been described as “probably the most widely read anti-democratic work of the post-Cold War era” (Gilley 2009, p. 120). It seems hopeful to believe that democratic legislators can be influenced by employing, what is perceived to be, anti-democratic rhetoric. If we intend to influence legislators we need to offer reasons they can accept. Beyond offering politically palatable reasons this immediately raises the question as to whether these reasons can be the abstract mathematical proofs of financial economics. Caplan’s thesis has also been challenged on the basis that he assumes what is true is determined by the consensus of what post-doctoral economists agree on, and this agreement is a consequence of the economists’ adherence to rational choice theory, which in turn posits that people should be objective utility maximisers. Our hypothesis on the moral content of the FTAP offers an alternative definition of what is rational to Caplan’s and provides a narrative that could make the abstract results of financial mathematics comprehensible to a broader public.

Given that the central thesis of this paper is concerned with reciprocity and Justice, we might expect that Rawls’ *A Theory of Justice* appears in the discussion. Because we rely on the Aristotelian framework we do not need Rawls. Another reason for not employing Rawls is given by Misak (2002, pp. 18–29) and is based on Rawls’ position that ‘Justice is political not metaphysical’. What this means is that Justice, reciprocity, cooperation, and so forth, are implicit in liberal democracies, but are not transcendentally true. This was not the Aristotelian position. The implication, as Misak makes clear, is that Rawlsians cannot say that the objective of cooperation is *right* (Misak 2002, p. 26). When Rubin quotes the libertarian Arthur C. Brooks’ emphatic statement that “The purpose of free enterprise is human flourishing, not materialism.” we can sense that Rubin wishes to cross Rawls’ ideological barrier and state that cooperation has precedence over competition. We justify our rejection of Rawls’ political Justice in favour of a transcendental conception of reciprocity on the basis of the evidence from the Ultimatum Game that indicates that the principle of reciprocity is universal in communities that engage in commercial exchange; it is not confined to liberal democracies. These results only emerged in the mid-1990s after Rawls had developed his theories.

Having presented arguments to address these concerns we then assume it is justified to claim that reciprocity is a key foundation of financial economics and offer an explanation for this fact: markets are centres of communicative action. Habermas developed the theory of communicative action to explain how democracies arrive at a consensus; we are interested in how markets arrive at a price and discuss the analogy. In the context of markets, reciprocity is one of the rules of discourse, alongside sincerity and charity, and develops in the practice of commerce to enable the achievement of social cohesion—the good internal to commerce. We are particularly interested in the role of mathematics in the price-setting process, and identify it as a mechanism of discourse. Specifically, the function of mathematics is to bring market participants to a shared understanding, it is not to determine a true price. Essentially we adopt a pragmatic meaning, rather than a propositional (truth-bearing) meaning for mathematics. There are implications of regarding markets as centres of communicative action on the practice and regulation of markets that we discuss in final part of “A Pragmatic Approach to Commerce” section with reference to: peer-to-peer lending and crowdfunding; order stuffing in high-frequency trading; and the LIBOR manipulation scandal.

Rubin’s discussion centres on cooperation and competition; we will claim that cooperation is central to financial economics by considering the concept of reciprocity, which is a feature of bipartite relations while cooperation is a more complex phenomenon involving many interactions. We we base our approach on Sahlins’ discussion of the significance of reciprocity in primitive economies [Sahlins 1972 (2003, Chap. 5)] and the proposition that reciprocity is the basis of human sociality presented in Henrich et al. (2004). Our use of ‘reciprocity’ in this paper is equivalent to Sahlins’ ‘balanced reciprocity’, which is associated with the ‘tribal sector’ where the degree of separation between agents is small. Trivers (1971) developed a model for how reciprocity evolves into cooperation in less connected networks based on the probability of repeated interactions that Axelrod and Hamilton (1981) adapted for the social sciences. Essentially, we assume that reciprocity is a feature of connected markets, where there is a likelihood of repeated interactions, and necessary for cooperation to emerge in less connected, more anonymous, markets. Competition comes into play when, for example, a buyer is offered prices by more than one seller. We shall focus on fairness in the reciprocal relationship between a buyer and seller, we shall only touch on the ‘fairness’ between sellers that enables competition by identifying sincerity, alongside reciprocity, as a norm of market discourse. This is particularly relevant in the huge, and relatively anonymous, LIBOR and foreign exchange markets that have been hit by scandals recently and in impersonal algorithmic trading. Another aspect of fairness that we touch upon is the fairness between agents of different status and we propose this is handled through the norm of charity. This is relevant if there is a difference in monetary or information wealth between agents and it is important in addressing the misselling of financial products, such as sub-prime mortgages or high interest loans.

The paper is structured as follows. “The Emergence of Probability” section begins with a description of medieval financial practice that highlights the sophistication and complexity of European commerce at the time. This is followed by a discussion of Scholastic analysis of commercial practice based on *Nicomachean Ethics*, this analysis is the genesis of mathematical probability. We then move on to explain the development of the mathematical theory of probability in the context of ethical investigations of commercial practice. “The Fundamental Theorem of Asset Pricing” section starts by explaining the development and significance of the FTAP. Then, building on the discussion in “The Emergence of Probability” section, it presents the main claim in an analysis of the FTAP as an ethical statement focusing on a correspondence between ‘no arbitrage’, ‘equal conditions’ and ‘martingale measures’. Acknowledging the ethical nature of modern probability we offer an interpretation of Ramsey’s Dutch Book argument as a re-statement of the Golden Rule: ‘Do unto others as you would have them do unto you’. The final part of “The Fundamental Theorem of Asset Pricing” section offers an explanation as to why the ethical nature of probability was obscured in the nineteenth century. We see this as an example of the process described in Adorno and Horkeimer’s *Dialectic of the Enlightenment* that is specifically concerned with a simultaneous ‘taming of chance’ (Hacking 1990) with a growing concern for problems of scarcity. In “A Pragmatic Approach to Commerce” section we employ Pragmatic philosophy to provide reasons to justify our claim, with the main justification coming from the empirical results of the Ultimatum Game. We then offer a meaning for the claim by employing some of Habermas’ ideas in *The Theory of Communicative Action* that were developed in response to the *Dialectic of the Enlightenment* and we relate these ideas to contemporary practice. We end this section by discussing some implications of linking our hypothesis to practice. Specifically, we hope that the public become more engaged with finance, rather than being passive consumers of financial products. Tangible consequences of our hypothesis would be regulatory support for mutual, non-profit seeking, mechanisms in finance and the inhibition of practices such as order-stuffing on automated exchanges.

## The Emergence of Probability

### Medieval Finance

From 1000 C.E. until about 1300 C.E. there was a rapid development of the economy in Western Europe as it evolved from an agriculturally based feudal society towards a commercially based bourgeois society, initially in Italy then, in the twelfth century, in North Western Europe. One physical manifestation of this change was the volume of coin circulating in the European economy, as the population doubled over the three hundred years, the amount of coin per person tripled (Pounds 1994, Chaps. 3 and 4; Kaye 1998, pp. 15–16; Nicholas 2006, p. 72).

#### Practice

Medieval European merchants, unlike their contemporaries in the Middle East, India or China, had to contend simultaneously with prohibitions on usury and the heterogeneity of currency. Muslim merchants had usury prohibitions but homogeneous currency, Indian and Chinese merchants had to (sometimes) deal with heterogeneous currencies but without the centralised religious prohibitions on usury.

Usury derives from the Latin *usus* meaning ‘use’, and referred to the charging of a fee for the use of money. Interest comes from the Latin *interesse* and originated in the Roman legal codes as the compensation paid if a contract was broken (Homer and Sylla 1996, p. 73). Shortly after 1200 the theologian, Peter the Chanter, argued that “a buyer or seller may be excused from usury if he exposes himself to the risk of receiving more or less” (Franklin 2001, pp. 263–264) and this idea that usury was absent in the presence of risk became firmly established in the thirteenth century.

The basic financial instrument at this time was the *census* that originated when ninth century monasteries guaranteed a fixed regular income in exchange for a donation of land. Censii developed to be written on the back of a diverse range of assets, including a craftsman’s labour, resembling modern day securitisation. In time ‘structured’ contracts emerged such that a borrower would receive a lump sum secured against the future cash-flow from an asset, *rente à prix d’argent*, without necessarily relinquishing ownership of the asset (Homer and Sylla 1996, pp. 75–76; Poitras 2000, pp. 31–33).

Modern structured finance was anticipated in the triple, or German, contract (*contractus trinus*), developed to fund long distance trade (Decock 2012). It involved a loan to fund the venture (the first contract); the transformation of the variable return of the venture into fixed cash-flow (the second contract); and an insurance contract to guarantee the fixed payment (the third contract). In terms of contemporary finance, this third contract is a Credit Default Swap and the whole contract has the type of structure of a Special Purpose Vehicle. This contract was declared illicit by the Catholic Church in 1586 on the basis that the lender received a risk-less return (Noonan 1957, pp. 209–220).

The heterogeneity of currency was a consequence of feudalism and the desire of magnates to assert their authority by issuing coin. The Italian peninsula had over twenty currencies, the Kingdom of France three, and each prince of the Holy Roman Empire would mint their own coin. Alfred Crosby describes the activities of a Tuscan merchant in supplying cloth to Venice from Mallorcan wool that involved at least five currencies (Crosby 1997, p. 201) . William Goetzman explains that as a consequence of the multitude of currencies, European medieval merchants “operated in a world of complete relativism” (Goetzmann 2004) while Crosby remarks that there was an “abstraction of Western merchants’ scale of value” and “no people were more obsessed with counting and counting and counting” (Crosby 1997, pp. 72, 74)

A solution to the problem of the complexity of Medieval commerce came in Fibonacci’s *Liber Abaci* first published in 1202, the initiant of financial economics (Crosby 1997, pp. 43–47; Fibonacci and Sigler 2003, Introduction). It was an immediate success and a second edition was produced in 1228, a remarkable feat in an age when books were hand copied. The text introduces Arabic/Hindu numerals and explains basic arithmetic over seven chapters. It then presents four chapters applying the theory by presenting cases on practical commercial problems. The text finishes with a more theoretical section on iterating to a solution of a problem (Fibonacci and Sigler 2003; Goetzmann 2004).

Before the *Liber Abaci*, European merchants, like their contemporaries across the globe, would have used an abacus to perform arithmetic calculations, and once a calculation had been made, it was recorded. The technologies described in the *Liber Abaci*, particularly Hindu numbers, meant that merchants could write down their calculation method, the algorithm, which could be copied and modified by others. Knowledge, in the form of best practice, could be created, distributed and improved.

*Abaco* or *rekoning* schools sprang up throughout Europe teaching apprentice merchants the techniques originating the *Liber Abaci*. The impact of these abaco schools was enormous, algebra became an important tool used by the large and influential community of Europeans and would provide the reservoir of mathematicians on which the scientific developments of the seventeenth century were built. The unique circumstances of medieval European commercial practice offer a solution to Needham’s question that asks why European technological development accelerated so much faster than Chinese after 1600 (Hadden 1994, Chap. 1; Fibonacci and Sigler 2003, Introduction; Heeffer 2008).

#### Theory

The science that emerged in Western Europe in the seventeenth century is distinctive in its use of mathematics to describe the laws of nature. The Greeks, and their Muslim successors, generally regarded ‘pure’ mathematics as being irrelevant to the sensible world while Chinese scientists used mathematics to calculate but not to describe (Crosby 1997, p. 16; Dear 2001, p. 164; Fara 2009, p. 53). Richard Hadden, Alfred Crosby and Joel Kaye have all argued that the ‘mathematisation’ of European science began with the synthesis of commercial practice and Scholastic ethics in the thirteenth and fourteenth centuries (Hadden 1994; Crosby 1997; Kaye 1998).

A key component of this synthesis was Aristotle’s *Nicomachean Ethics* that addresses how an individual can live as part of a community and it discusses economics in Book V in the context of the virtue of Justice. Aristotle distinguishes economic justice into two main classes, distributive (in V.3) and restorative (or corrective, in V.4). Distributive justice is concerned with the distribution of common goods by a central authority in proportion to the recipients’ worth and is determined by equating Geometric Proportions. Restorative justice applies in cases where the parties are considered to be equal but there has been an erroneous allocation which is corrected by equating Arithmetic Proportions (Kaye 1998, pp. 41–43; Broadie and Rowe 2011, pp. 1130b30–31a5)

Our case is built on the discussion of ‘justice in associations for exchange’ in V.5, which has proved problematic for commentators over the centuries (Judson 1997). Justice in exchange is distinguished from distributive and restorative justice by being characterised by proportionate equality. Fundamental to this principle is that there is an *equality* of goods exchanged, “there is no giving in exchange”, since it is a reciprocal arrangement, nor is there a corrective aspect to exchange. Reciprocity in exchange is essential in binding society together, it is important for social cohesion not in order to generate a profit (Kaye 1998, p. 51; Broadie and Rowe 2011, pp. 1133a15–30). These points are explained in detail by Judson (1997), who begins his article with a statement relevant to our discussion

Aristotle’s concern is solely with an ethical question, namely ‘What is the basis of *fairness* in the exchange of goods?’, and not with economic analysis of any sort, even as a subordinate part of ethical enquiry. (Judson 1997, pp. 147–148, emphasis in the original)

Aristotle’s argument is ethical and mathematical and justice in exchange is concerned with fairness and equality in order to establish social cohesion. It is not an economic analysis in the sense of the modern understanding of addressing a problem of efficiently distributing scarce resources.

It is remarkable that Aristotle approached the problem mathematically since he rarely applied mathematics to the sensible world elsewhere (Hadden 1994, p 75; Crosby 1997, p. 13; Broadie and Rowe 2011, pp. 1094b15–28). Aristotle realised that if there was to be equality then

everything that is exchanged must be somehow comparable. This is the role that is fulfilled by currency [*nomisma*], so that it becomes, in a way, an intermediate. (Broadie and Rowe 2011, pp. 1133a19–20)

These lines are significant for two reasons. Firstly the word *nomisma* for currency/money is related to the concepts of custom and law, not to ‘labour and expenses’. Second, ‘intermediate’ is in the sense of a mediator between two objects, rather than simply as a token, which is a more modern interpretation. Furthermore, Aristotle defined the quality that money measured by the word *chreia*, which was initially translated to *opus* (work), but was later corrected to *indigentia* (need) (Kaye 1998, pp. 68–70). This is important because it demonstrates that Aristotle and the Scholastics viewed money as a social construction binding society by allowing an exchange based on need, rather than as a simple commodity facilitating the exchange of sensible quantities, such as labour and expenses.

The significance of the Scholastic analysis to the development of science was that when Aristotle discussed measurement in the context of physics he argued that the measure shared the ‘substance’ of the measured; this meant that wine was incommensurable with cloth, time incommensurable with space. The Scholastics recognised that money was a very special measure; it applied to all goods in a market, and only occasionally shared the substance of the goods. This insight enabled them to revolutionise the concept of measurement, in a way that contemporary Muslim scholars did not, and allowed Jean Buridan to identify the concept of inertia (Boyer and Merzbach 1991, pp. 263–268; Crosby 1997, pp. 67–74; Kaye 1998, pp. 65–70).

Out of Aristotle’s discussion of market exchange, Scholastics developed the concept of the ‘Just Price’, which has been the subject of considerable modern debate. For example, Raymond de Roover (1958) argues against viewing the Just Price in a Marxist, labour theory of value, sense but rather as the market price, in a neo-classical, liberal sense. However, neither of these modern positions corresponds to how the Scholastics viewed the concept. The interpretation of the Just Price we shall employ, based on the Scholastic attitudes to Aristotle’s description of exchange, is the one discussed by Monsalve (2014). The Just Price represents an “intellectual construct: an ideal price that guarantees equality in exchange” and that it represents a mathematical ‘medium’ or a ‘mean’.

Monsalve points out that Scholastic analysis was conducted in a definite moral frame of reference and so the Just Price “could not refer indiscriminately to whatever price might be obtained in the market” (Monsalve 2014, p. 8, quoting Langholm). This aspect was discussed in detail by the Scholastics prompted by a question ‘Whether the seller is bound to state the defects of the thing sold?’ posed by Aquinas (1947, II, ii, qu. 77, art. 3, ad. 4). Specifically Aquinas addresses a problem originating in Stoic philosophy relating to the conduct of a merchant carrying a supply of food to a starving country. The merchant knows that they are the first of a number of merchants bringing food, the question is, should he sell the food at the high ‘market’ price or a lower price based on his knowledge.

Kaye makes the point that Aquinas separates the Just Price, determined by divine law, from the ‘market price’, established by men, and explains that if the Just Price equated with the market price then an “individual’s responsibility in economic activity is effectively eliminated” (Kaye 1998, p. 98). Despite recognising this distinction, the answer from Aquinas is a little surprising. Aquinas observes that the merchant may *believe* that there are more grain shipments on the way, but does not *know*: the future is uncertain. On the basis that there is no certainty, and on the authority of Peter the Chanter, the merchant may charge the going market price, making an excessive but nevertheless legitimate profit, though it would be more virtuous to charge the lower price. Aquinas’ conclusion is surprising because it suggests the merchant can be insincere in his actions.

Aquinas’ argument was criticised by Pierre Jean Olivi, a leader of the ‘Spiritual Franciscans’. The Spiritual Franciscans argued that the vow of poverty meant monks should limit their use of property, *usus pauper*, a more severe restriction than just not owning property. As a consequence of this extreme position Olivi was posthumously condemned as a heretic in 1326, hindering the subsequent transmission of his thought. The Franciscans, unlike the empirical rationalist Dominicans such as Thomas Aquinas, were fideists and this philosophical approach meant that Olivi argued that the metaphysical probability of more grain arriving had a certain reality, which Aquinas was ignoring (Kaye 1998, p. 121). Olivi said

The judgement of the value of a thing in exchange seldom or never can be made except through conjecture or probable opinion, and not so precisely, or as if understood and measured by one invisible point, but rather as a fitting latitude within which the diverse judgements of men will differ in estimation. (Kaye 1998, p. 124).

This distinction is essential in demarcating the Just Price, an imprecise abstraction, from the market price, which is observed at a fixed point (Monsalve 2014, Sect. 3.2.1).

Olivi seems to have interacted with merchants and been a close observer of markets and considered a number of aspects of commerce including the problem of usury (Franklin 2001, p. 265). Based on the principle that a lender could charge a borrower compensation for a loss (*interesse*) Olivi recognised that borrowers should compensate lenders for the ‘probable profit’ they could earn by employing capital elsewhere. Fair exchange was a question of restoring ‘probable equivalence’, not of precise equality (Kaye 1998, p. 119; Franklin 2001, pp. 265–267). As part of this argument Olivi commented that a valuation did not only depend on ‘need’ but also on a good’s scarcity, usefulness and desirability. Since both need and desirability are subjective, different people will value the same good differently and based on these ideas, Olivi was able also to explain the ‘value paradox’ (Rothbard 1996, pp. 60–61; Kaye 1998, pp. 123–124). Ultimately, according to James Franklin, Olivi thought of probability as a trade-able entity, and so could be quantified (Franklin 2001, pp. 266–267).

### The Science of Conjecture

The Science of Conjecture, or Probability, is the rational method for dealing with uncertainty. Aristotle classified events into three types: certain events determined by specific causes; probable events that usually happened; and unpredictable events, including games of chance, not amenable to science (Hald 1990, p. 30). The development of Probability over the past five hundred years has been concerned principally with reducing the scope of those events ‘not amenable to science’ in support of the Cartesian programme to place knowledge on indubitable foundations (Grayling 2005, pp. 281–285).

While Olivi and merchants developed the idea of probability in relation to commercial exchange and jurists and theologians addressed questions of proof, the concept of quantifying chance did not fully materialise until the mid-sixteenth century with Cardano’s *Liber de Ludo Alea*. Ian Hacking has remarked Hacking (1984, Chap. 1) that the emergence of the concept of absolute chance was late; however, this identification of mathematical probability in the context of finance precedes both Descartes’ introduction of absolute space (Cartesian co-ordinates) and Newton’s of absolute time.

Up until the 1950s, and a re-assessment of his work by Ore (1953), Cardano’s contribution to probability theory had been widely ignored. In the context frequentist interpretations of probability, that dominated the nineteenth and early twentieth centuries, it was seen as incoherent. More recently, Bellhouse (2005) has re-evaluated the *Liber* looking at it as a humanist philosophical text, not as a mathematical document, based on the fact that Cardano, himself, did not list it as one of his mathematical works. Bellhouse’s hypothesis is that in the *Liber* Cardano is trying to establish under what grounds gambling can be considered ethical in the context of *Nicomachean Ethics*.

Cardano latches on to the idea that Justice is equivalent to equality and argues that in dice games ‘equality’ was established by counting the ways a player could win and comparing that number to the ways a player would lose. On this basis the ‘chance’ of winning could be deduced, and if the stakes did not match the chances, the gamble was unjust. Summarising his findings he states, “a just gamble is one between willing and knowledgeable players”, making an explicit association between science and ethics. Almost immediately after coming to these ethical conclusions, Cardano observes that

These facts contribute a great deal to understanding but hardly anything to practical play (David 1998, p. 58) quoting from Chapter 9 of the *Liber*)

since they offer nothing to help forecast the outcome of the dice throw.

One problem Cardano considered was the so-called *Problem of Points* which appears in a text by Pacioli and is based on the following situation:

Two players, *F* and *P*, are playing a game based on a sequence of rounds, and each round consists of, for example, the tossing of a fair coin. The winner of the game is the player who is the first to win 7 rounds, and they will win 80 francs.

The *Problem of Points* is how the 80 francs should be split if the game is forced to end after *F* has won 5 rounds while *F* has won 4. Edith Dudley Sylla notes that the *Problem* comes from the abaco tradition of using ‘stories’ to give examples of how to solve problems in commercial arithmetic. In this case the *Problem of Points*, the story represents the case of how the capital tied up in a business partnership should be divided if the venture has to finish prematurely (Sylla 2003).

Pacioli’s solution was statistical, the pot should be split 5:4. Cardano recognised this was absurd since it would give a manifestly unfair result if the game ended after one round out of a hundred or when *F* had 99 wins to *P*’s 90. Cardano makes the point that the correct solution would be arrived at by considering what would happen in the future, it had to be forward-looking, in particular, it had to account for what ‘paths’ the game would follow. Despite this insight, Cardano’s solution was still wrong, and the correct solution was provided by Pascal and Fermat in their correspondence of 1654.

The Pascal–Fermat solution to the *Problem of Points* is widely regarded as the starting point of mathematical probability. The pair (it is not known exactly who) realised that when Cardano calculated that *P* could win the pot if the game followed the path *PP* (i.e. *P* wins and *P* wins again) this actually represented four paths, *PPPP*, *PPPF*, *PPFP*, *PPFF*, for the game. It was the players’ ‘choice’ that the game ended after *PP*, not a feature of the game itself and this represents an early example of mathematicians disentangling behaviour from problem structure. Calculating the proportion of winning paths would come down to using the Arithmetic, or Pascal’s, Triangle—the Binomial distribution. Essentially, Pascal and Fermat established what would today be recognised as the Cox–Ross–Rubenstein formula (Cox et al. 1979) for pricing a digital call option.

The Pascal–Fermat correspondence was private, the first textbook on probability was written by Christiaan Huygens in 1656. Huygens had visited Paris in late 1655 and had been told of the *Problem of Points*, but not of its solution (David 1998, p. 111); Hald 1990, p. 67), and on his return to the Netherlands he solved the problem for himself and produced the first treatise on mathematical probability, *Van Rekeningh in Speelen van Geluck* (‘On the Reckoning of Games of Chance’) in 1657.

In *Van Rekeningh* Huygens starts with, what is essentially, an axiom,

I take as fundamental for such [fair] games that the chance to gain something is worth so much that, if one had it, one could get the same in a fair game, that is a game in which nobody stands to lose.(Hald 1990, p. 69)

Probability is defined by equating future gain with present value in the context of ‘fair’ games.

In the 1670’s probability theory developed in the context of Louis XIV’s *appartements du roi*, thrice weekly gambling events that have been described as a ‘symbolic activity’ not unlike *potlach* ceremonies that bind primitive communities (Kavanagh 1993, pp. 31–42). This mathematical analysis of an important social activity stimulated the publication of books describing objective, or frequentist, probability. The empirical, frequentist, approach began to dominate the mathematical treatment of probability following the claimed ‘defeat’, or ‘taming’, of chance by mathematics with the publication of Montmort’s *Essay d’Analyse sur les Jeux de Hazard* (‘Analytical Essay on Games of Chance’) of 1708 and De Moivre’s *De Mensura Sortis* (‘The Measurement of Chance’), of 1711 developed in *The Doctrine of Chances* of 1718 (Bellhouse 2008). These texts were developed in response to ‘fixed odds’ games of chance rather than in the analysis of commercial contracts. *The Doctrine* was the more influential, introducing the Central Limit Theorem, and by 1735 it was believed that there was no longer a class of events that were ‘unpredictable’ (Bellhouse 2008).

Around 1684 James Bernoulli had begun working on problems in probability and between 1700 and his death in 1705 he worked on *Ars Conjectandi* (‘The Art of Conjecturing’), a title that emphases the practical rather than theoretical nature of conjecture, which was published posthumously in 1713. The *Ars* is made up of four parts, a commentary on Huygens’ *Van Rekeningh*, original work on calculating permutations and combinations, applications of these ideas to games of chance and finally the application of the ideas to “civil, moral and economic affairs” (Hald 1990, p. 224).

While the first three sections of the *Ars* are un-controversial, the final section is both the most significant and has proved problematic. Bernoulli, having discussed objective probability at length introduces the epistemic, or subjective, definition of probability as “a degree of certainty”. Anders Hald notes that this is “revolutionary” because Bernoulli is applying mathematics to propositions, not just to events (Hald 1990, p. 225). This section of the *Ars* is significant in that it introduces what would become known as the ‘Law of Large Numbers’, which can be summarised as collecting a large amount of data will improve the accuracy of an observation—providing the system was stationary (Hald 1990, p. 225). The section is problematic because Bernoulli considered situations where the sum of probabilities could be greater than one (Sylla 2006, p. 27). This is impossible if probability is calculated as relative frequency.

Sylla compared Bernoulli’s work to that of Huygens’ and other contemporaries, de Witt and de Moivre, in the process of translating the *Ars* and concluded that

equity among associates or partners rather than probabilities in the sense of relative frequencies provided the foundation for the earliest mathematical probability theory. (Sylla 2006, p. 13)

and that

While traditional histories of mathematical probability start with Pierre Fermat, Pascal and Huygens because they give what are from the modern point of view correct frequentist solutions to the problems of division and expectations in games of chance ...the foundations of Huygens’ method (…) was not chance (frequentist probability), but rather *sors* (expectation) in so far as it was involved in implicit contracts and the just treatment of partners. (Sylla 2006, p. 28)

In the sixteenth and seventeenth centuries the motivation for the development of probability was in the ethical analysis of commercial contracts where Justice, balanced reciprocity or ‘fairness’ dominated. The later Empirical approach to probability, based on observing relative frequencies, emerged out of the simpler analysis of games of chance in the context of fixed odds.

The case that Huygens was working in the context of Virtue Ethics is enhanced by recognising the difficulty he had in translating *Van Rekeningh* into Latin (Hacking 1984, pp. 93–94). Huygens struggled to translate the Dutch word *kans* (‘chance’, ‘lot’), which would normally be translated as *sors*, and eventually he, or his editor van Schooten, chose *expectatio*, giving the English term ‘expectation’ (in the mathematical sense). However, Huygens had considered using the Latin word *spes* (Hacking 1984, p. 95) which was the term for the virtue ‘Hope’. In French, *espérance* is used when referring to mathematical expectation, reflecting this debate. The Dutch, who following Stevin’s focus on teaching mathematics in the vernacular, use their own terms in mathematics, in this case the equivalent is *verwachting*: hope, promise, expectation, forecast, prognosis.

Sylla also observes that *The Port Royal Logic*, a significant influence on Pascal, notes that “because the house takes part of the stakes, lotteries are manifestly unfair” and seventeenth century mathematicians recognised a distinction between actual gambles, involving transaction costs, and idealised, frictionless, markets, suitable for the mathematical study by academics (Sylla 2003, p. 327).

## The Fundamental Theorem of Asset Pricing

The Fundamental Theorem of Asset Pricing consists of two statements, (e.g. (Shreve 2004, Section 5.4))

1. A market admits no arbitrage, if and only if, the market has a martingale measure.
2. Every contingent claim can be hedged, if and only if, the martingale measure is unique.

### The Context of the FTAP

The FTAP emerged between 1979 and 1983 (Harrison and Kreps 1979; Harrison and Pliska 1981, 1983) as Michael Harrison sought to establish a mathematical theory underpinning the Black–Scholes–Merton (BSM) equation for pricing options, which was introduced in 1973.

In the late 1960s, Fischer Black and Myron Scholes worked as investment consultants and one of the problems the pair addressed was the valuation of ‘warrants’, options bundled with bonds. Black was an applied mathematician who had worked in consultancy for Jack Treynor around the time that Treynor developed his version of the Capital Asset Pricing Model (CAPM). Scholes had studied for a doctorate under Eugene Fama looking at risk-reward in the context of efficient markets (Scholes 1972). Black tackled the problem of pricing warrants as an applied mathematician: the value of the warrant would be a function of the underlying asset’s price and amenable to the type of calculus that had been employed since Newton and Leibnitz. Scholes approached the problem from a financial perspective: the risk of holding a warrant could be removed by holding a complementary (short) position in the underlying asset, by hedging. What Scholes did not know was how to establish the size of the hedging portfolio, but when he discussed this with Black they realised the solution was in the slope of the function relating the warrant price and asset price, a result that had been anticipated by Thorp and Kassouf (MacKenzie 2008, pp. 130–131).

Simultaneously, Robert C. Merton, who had studied advanced engineering mathematics before becoming a student of Paul Samuelson, was considering the problem of pricing warrants from a different perspective. Samuelson had never accepted Markowitz’s criterion of trading the expected returns of a portfolio against the variance of returns (Samuelson 1970), which was a foundation of CAPM and Scholes’ work, so Merton tackled the problem of valuing warrants by maximising expected utility employing the stochastic calculus that had become important in aeronautical and electronic engineering. This work was published in 1969 (Samuelson 1969; Merton 1969).

Despite the fact that Black never liked Merton’s highly mathematical technique, Scholes discussed their work with Merton in 1970. Merton saw how the Black–Scholes approach of hedging could be incorporated into his own continuous time models, removing the need to employ an arbitrary utility function in solving the pricing problem. Merton showed that a portfolio made up of: a single warrant, or an option; a hedging position in the risky underlying asset; and a funding position in the riskless bank account, would offer the same, certain, return as the initial cost of the portfolio deposited in the riskless bank account. It seemed that both subjectivity and risk had been removed from the pricing problem.

In October 1970 Black and Scholes submitted their work to the *Journal of Political Economy* and then the *Review of Economics and Statistics*, but it was rejected without review, on the basis that there was not enough economics in it. The paper was only published by the *Journal of Political Economy* (Black and Scholes 1973) after the intervention of influential academics and shortly after the opening of the Chicago Board Options Exchange (Bernstein 1998, pp. 314–315; MacKenzie 2008, pp. 133–136). Merton published his approach almost simultaneously (Merton 1973).

When BSM was being developed option pricing was a relatively unimportant activity. Gambling legislation in the United States meant that options were only traded on ‘deliverable’ assets, principally agricultural commodities, and these markets were stagnant (MacKenzie 2008, pp. 142–145). However, following the ‘Nixon Shock’ of August 1971, the Bretton Woods system of fixed exchange rates collapsed and in the aftermath, interest rates, exchange rates and commodity prices became much more volatile. Options, which have been a feature of financial practice since the seventeenth century and were widely traded before the suspension of the European financial markets during the First World War (Nelson 1904), re-emerged as a tool to insure against volatile asset prices.

Despite the financial rational for options, their legitimacy with regard to gambling legislation was still ambiguous. The introduction of BSM delivered a mathematical equation that defined the price of an option in terms of known, in the sense of statistically certain, parameters making their valuation appear deterministic. Trading in options could not be gambling, given that there was no speculation in their valuation. Donald MacKenzie reports the view of the legal counsel to the Chicago Board of Trade at the time, Burton Rissman

Black–Scholes was what really enabled the exchange to thrive …we were faced in the late 60s—early 70s with the issue of gambling. That fell away, and I think Black–Scholes made it fall away. It wasn’t speculation or gambling it was efficient pricing. (MacKenzie 2008, p. 158)

Both the Black–Scholes and Merton approaches to pricing options involved heuristic arguments, they were ‘engineering solutions’. Harrison sought to establish a rigorous option pricing ‘theory’ to support the range of mathematical models developed on the back of the explosion in derivatives markets (MacKenzie 2008, pp. 140–141). Harrison, and his colleagues, were successful in their mission and opened finance to investigation by pure mathematicians (e.g. Schachermayer 1984; Delbaen and Schachermayer 1994; Delbaen and Schachermayer 1998) and by 2000, any mathematician working on asset pricing would do so within the context of the FTAP.

The FTAP is not well known outside the academic field of financial mathematics. Practitioners focus on the models that are a consequence of the Theorem while social scientists focus on the original Black–Scholes–Merton approach as an exemplar. Even before the market crash of 1987 practitioners were sceptical as to the validity of the prices produced by their models (Miyazaki 2007, pp. 409–410; MacKenzie 2008, p. 248; Haug and Taleb 2011) and today the original Black–Scholes equation is used to measure market volatility, a proxy for uncertainty, rather than to ‘price’ options.

However, the status of the Black–Scholes model as an exemplar in financial economics has been enhanced following the development of the FTAP. Significantly, the theorem unifies different approaches in financial economics. The clearest example of this synthesis was that in the course of the development of the FTAP it was observed that a mathematical object, the Radon–Nikodym derivative, which is related to the stochastic calculus Merton employed, involved the market-price of risk (Sharpe ratio), a key object in CAPM that Black used. Without the FTAP the two approaches are incongruous (MacKenzie 2003a, p 834). Overall, as will be discussed in full in the next section, the FTAP brings together: Merton’s approach employing stochastic calculus advocated by Samuelson; CAPM, developed by Treynor and Sharpe; martingales, a mathematical concept employed by Fama in the development of the Efficient Markets Hypothesis; and the idea of incomplete markets, introduced by Arrow and Debreu.

The synthesis by the FTAP of a ‘constellation of beliefs, values, techniques’ represented a Kuhnian paradigm for financial economics focused on the Black–Scholes–Merton approach to pricing options. To paraphrase Tait

A mathematical proposition is about a certain structure, financial markets. It refers to prices and relations among them. If it is true, it is so in virtue of a certain fact about this structure. And this fact may obtain even if we do not or cannot know that it does. (Tait 1986, p 341)

In this sense, the FTAP confirmed the ‘truth’ of many of the core concepts of financial economics in the mid 1990s.

### An Ethical Analysis of the FTAP

The FTAP is a theorem of mathematics, and the use of the term ‘measure’ in its statement places the FTAP within the theory of probability formulated by Kolmogorov in 1933 (1956). Kolmogorov’s work took place in a context captured by Bertrand Russell, who in 1927 observed that

It is important to realise the fundamental position of probability in science. …As to what is meant by probability, opinions differ. (Russell 1927 (2009, p. 301)

In the 1920s the idea of randomness, as distinct from a lack of information, was becoming substantive in the physical sciences (Hacking 1990, p. 1; von Plato 1994, pp. 147–157). In the social sciences, Frank Knight argued that uncertainty was the only source of profit (Knight 1921 (2006, III.VII.1–4) and the concept was pervading John Maynard Keynes’ economics (Mizuhara and Runde 2004; Skidelsky 2009, pp. 84–88).

Two mathematical theories of probability had become ascendant by the late 1920s. Richard von Mises (brother of the Austrian economist Ludwig) von Mises (1957) attempted to lay down the axioms of classical probability within a framework of logical positivism, the ‘frequentist’ or ‘objective’ approach. To counter-balance von Mises, the Italian actuary Bruno de Finetti presented a very different approach, characterised by his claim that “Probability does not exist” because it was only an expression of the observer’s view of the world. This ‘subjectivist’ approach was closely related to the position taken by Frank Ramsey who developed an argument against Keynes’ interpretation of probability presented in the *Treatise on Probability* (Ramsey 1931; Ramsey and Mellor 1980; Davis 2004; Edgington 2012).

Kolmogorov addressed the diversity of mathematical probability by generalising so that all were examples of abstract ‘measures’ satisfying certain axioms. In doing this, a random variable became a function while an expectation was an integral: probability became a branch of Analysis, not Statistics.

Von Mises criticised Kolmogorov’s generalised framework as un-necessarily complex (von Mises 1957, p. 99) while the statistician Maurice Kendall argued that abstract measure theory failed “to found a theory of probability as a branch of scientific method” (Kendall 1949, p. 102). More recently the physicist Edwin Jaynes champions Leonard Savage’s subjectivist Bayesianism as having a “deeper conceptual foundation which allows it to be extended to a wider class of applications, required by current problems of science” (Jaynes 2003, p. 655).

The objections to measure theoretic probability for empirical scientists can be accounted for as a lack of physicality. Frequentist probability is based on the act of counting; subjectivist probability is based on a flow of information, which following Claude Shannon, is now an observable entity in empirical science. Measure theoretic probability is based on abstract mathematical objects unrelated to sensible phenomena. However, the generality of Kolmogorov’s approach made it flexible enough to handle problems that emerged in physics and engineering during the Second World War and his approach became widely accepted after 1950 because it was practically useful.

In the context of the first statement of the FTAP, a ‘martingale measure’ can be understood as a probability measure, usually labelled $\mathbb {Q}$, such that the (real, rather than nominal) price of an asset today, $X_0$, is the expectation, using the martingale measure, of its (real) price in the future, ${X}_{T}$. Formally,

$$X_0 =\mathbb {E}_\mathbb {Q}[ {X}_{T}].$$

The abstract probability distribution $\mathbb {Q}$ is defined so that this equality exists, not on any empirical information of historical prices or subjective judgement of future prices. The only condition placed on the relationship that the martingale measure has with the ‘natural’, or ‘empirical’, probability measures, usually assigned the label $\mathbb {P}$, is that they agree on what is possible.

The term ‘martingale’ in this context derives from doubling strategies in gambling and it was introduced into mathematics by Jean Ville in a development of von Mises work. The idea that asset prices have the martingale property was first proposed by Mandelbrot (1966) in response to an early formulation of Eugene Fama’s Efficient Market Hypothesis (EMH) (Fama 1965), the two concepts being combined by Fama (1970). For Mandelbrot and Fama the key consequence of prices being martingales was that the current price was independent of the future price and technical analysis would not prove profitable in the long run. In developing the EMH there was no discussion on the nature of the probability under which assets are martingales, and it is often assumed that the expectation is calculated under the natural measure. While the FTAP employs modern terminology in the context of positivist economic value-neutrality, the idea of equating a current price with a future, uncertain, payoff would have been understood by Olivi and obvious to Huygens, both working in an explicitly ethical framework.

The other technical term in the first statement of the FTAP, arbitrage, has long been used in financial mathematics. In Chapter 9 of the *Liber Abaci* Fibonacci discusses ‘Barter of Merchandise and Similar Things’,

20 arms of cloth are worth 3 Pisan pounds and 42 rolls of cotton are similarly worth 5 Pisan pounds; it is sought how many rolls of cotton will be had for 50 arms of cloth. (Fibonacci and Sigler 2003, p. 180)

In this case there are three commodities, arms of cloth, rolls of cotton and Pisan pounds, and Fibonacci solves the problem by having Pisan pounds ‘arbitrate’, or ‘mediate’ as Aristotle might say, between the other two commodities. Over the centuries this technique of pricing through arbitration evolved into the Law of One Price: if two assets offer identical cash flows then they must have the same price. This was employed by Jan de Witt in 1671 when he solved the problem of pricing life annuities in terms of redeemable annuities, based on the presumption that

the real value of certain expectations or chances of objects, of different value, should be estimated by that which we can obtain from as many expectations or chances dependent on one or several equitable contracts. (Sylla 2003, p. 313, quoting De Witt)

In 1908 Vincent Bronzin published a text which discusses pricing derivatives by ‘covering’, or hedging, them with portfolios of options and forward contracts employing the principle of ‘equivalence’ (Zimmermann and Hafner 2007). In 1965 the mathematicians, Edward Thorp and Sheen Kassouf, combined the Law of One Price with basic techniques of calculus to identify market mispricing of warrant prices and in 1967 they published their methodology in a best-selling book, *Beat the Market*.

Within neo-classical economics, the Law of One Price was developed in a series of papers between 1954 and 1964 by Kenneth Arrow, Gérard Debreu and Lionel MacKenzie in the context of general equilibrium, in particular the introduction of the Arrow Security, which, employing the Law of One Price, could be used to price any asset (Arrow 1964). It was on this principle that Black and Scholes believed the value of the warrants could be deduced by employing a hedging portfolio. By introducing their work with the statement that “it should not be possible to make sure profits” (Black and Scholes 1973) they were invoking the arbitrage argument, which had an eight hundred year history.

In the context of the FTAP, ‘an arbitrage’ has developed into the ability to formulate a trading strategy such that the probability, under a natural or martingale measure, of a loss is zero, but the probability of a positive profit is not. This definition is important following Hardie’s criticism of the way the term is applied loosely in economic sociology, and elsewhere (Hardie 2004). The important point of this definition is that, unlike Hardie’s definition (Hardie 2004, p. 243), there is no guaranteed (strictly positive) profit.

To understand the connection between the financial concept of arbitrage and the mathematical idea of a martingale measure, consider the most basic case of a single asset whose current price, $X_0$, can take on one of two (present) values, ${X}_T^D<{X}_T^U$, at time $T>0$, in the future. In this case an arbitrage would exist if $X_0\le X_T^D<X_T^U$: buying the asset now, at a price that is less than or equal to the future pay-offs, would lead to a possible profit at the end of the period, with the guarantee of no loss. Similarly, if $X_{T}^{D}<X_{T}^{U}\le X_{0}$, short selling the asset now, and buying it back would also lead to an arbitrage. So, for there to be no arbitrage opportunities we require that

$$X_{T}^{D}< X_{0} < X_{T}^{U}.$$

This implies that there is a number, $0< q < 1$, such that

$$X_0 = X_{T}^{D} + q\,(X_{T}^{U} - X_{T}^{D})=q\,X_{T}^{U} +(1-q)X_{T}^{D}.$$

The price now, $X_{0}$, lies between the future prices, $X_{T}^{U}$ and $X_{T}^{D}$, in the ratio $q:(1-q)$ and represents some sort of ‘average’. The first statement of the FTAP can be interpreted simply as “the price of an asset must lie between its maximum and minimum possible (real) future price”.

If $X_{0}<X_{T}^{D}\,\le\, X_{T}^{U}$ we have that $q<0$ where as if $X_{T}^{D}\,\le X_{T}^{U}< X_{0}$ then $q>1$, and in both cases *q* does not represent a probability measure which by Kolmogorov’s axioms, must lie between 0 and 1. In either of these cases an arbitrage exists and a trader can make a riskless profit, the market involves *‘turpe lucrum’*. This account gives an insight as to why James Bernoulli, in his moral approach to probability, considered situations where probabilities did not sum to 1, he was considering problems that were pathological not because they failed the rules of arithmetic but because they were unfair.

It follows that if there are no arbitrage opportunities then quantity *q* can be seen as representing the ‘probability’ that the $X_{T}^{U}$ price will materialise in the future. Formally

$$\begin{aligned} X_{0} = &q\,X_{T}^{U} +(1-q)X_{T}^{D}\\ \equiv&\mathbb {E}_\mathbb {Q}\big [ {X}_T \big ]. \end{aligned}$$

The connection between the financial concept of arbitrage and the mathematical object of a martingale is essentially a tautology: both statements mean that the price today of an asset must lie between its future minimum and maximum possible value.

This first statement of the FTAP was anticipated by Ramsey in 1926 when he defined ‘probability’ in the sense of ‘a degree of belief’ and argued that a standard way of measuring ‘degrees of belief’ is through betting odds (Ramsey 1931, p. 171). On this basis he formulates some axioms of probability, including that a probability must lie between 0 and 1 (Ramsey 1931, p. 181). He then goes on to say that

These are the laws of probability, ...If anyone’s mental condition violated these laws, his choice would depend on the precise form in which the options were offered him, which would be absurd. He could have a book made against him by a cunning better and would then stand to lose in any event. (Ramsey 1931, p. 182)

This is a concrete practical argument, rather than an abstract theoretical one, that identifies the absence of the martingale measure with the existence of arbitrage and today it forms the basis of the standard argument as to why arbitrages do not exist: if they did the, other market participants would bankrupt the agent who was mispricing the asset. This has become known in philosophy as the ‘Dutch Book’ argument and, as a consequence of the value-neutrality that dominates modern science, is often presented as a ‘matter of fact’. However, if we ignore value-neutrality and accept that probability has an ethical dimension then the Dutch book argument is an alternative of the ‘Golden Rule’—“Do to others as you would have them do to you.”—it is infused with the moral concepts of fairness and reciprocity (Wattles 1996; Hájek 2008).

Ramsey goes on to make an important point

Having any definite degree of belief implies a certain measure of consistency, namely willingness to bet on a given proposition at the same odds for any stake, the stakes being measured in terms of ultimate values. Having degrees of belief obeying the laws of probability implies a further measure of consistency, namely such a consistency between the odds acceptable on different propositions as shall prevent a book being made against you. (Ramsey 1931, pp. 182–183)

Ramsey is arguing that an agent needs to employ the same measure in pricing all assets in a market, and this is the key result in contemporary derivative pricing. Having identified the martingale measure on the basis of a ‘primal’ asset, it is then applied across the market, in particular to derivatives on the primal asset but the well-known result that if two assets offer different ‘market prices of risk’, an arbitrage exists. This explains why the market-price of risk appears in the Radon–Nikodym derivative and the Capital Market Line, it enforces Ramsey’s consistency in pricing.

The second statement of the FTAP is concerned with incomplete markets, which appear in relation to Arrow-Debreu prices. In mathematics, in the special case that there are as many, or more, assets in a market as there are possible future, uncertain, states, a unique pricing vector can be deduced for the market because of Cramer’s Rule. If the elements of the pricing vector satisfy the axioms of probability, specifically each element is positive and they all sum to one, then the market precludes arbitrage opportunities. This is the case covered by the first statement of the FTAP.

In the more realistic situation that there are more possible future states than assets, the market can still be arbitrage free but the pricing vector, the martingale measure, might not be unique. An agent should still be consistent in selecting which particular martingale measure they choose to use, but another agent might choose a different measure, such that the two do not agree on a price. In the context of the Law of One Price, this means that we cannot hedge, replicate or cover, a position in the market, such that the portfolio is riskless. The significance of the second statement of the FTAP is that it tells us that in the sensible world of imperfect knowledge and transaction costs, a model within the framework of the FTAP cannot give a precise price. When faced with incompleteness in markets, agents need alternative ways to price assets and behavioural techniques have come to dominate financial theory. This feature was realised in *The Port Royal Logic* when it recognised the role of transaction costs in lotteries.

### The Dialectic of Enlightenment

We present the case that the subsistence of the FTAP is reciprocity, alternatively Justice characterised by equality in exchange, colloquially fairness. The pre-history of mathematical probability lies in Olivi’s examination of commercial exchange in the context of Aristotle’s *Ethics*. The formal introduction of mathematical probability in the seventeenth century is in the ethical analysis of commercial contracts in the context of ‘fair’ pricing. However, during the nineteenth century the moral injunction not to engage in *turpe lucrum*, through the practice of arbitrage, becomes highly technical, and ethically neutral. In the process the essence of reciprocity in the FTAP becomes obscured. This immediately raises the question as to why, or how, did the association disappear in the nineteenth century.

The idea that commerce improved society was prevalent throughout the eighteenth century in the *doux-commerce* thesis. A 1704 technical text on commerce argues “Commerce attaches [men] to one another through mutual utility”; while in *The Rights of Man* (1792) Thomas Paine writes “commerce is a pacific system, operating to cordialise mankind”. In the intervening years Montesquieu, Hume, Condorcet and Adam Smith all agreed that commerce was a powerful civilising agent, promoting honesty, industriousness, probity, punctuality, and frugality, in contrast to the excesses of absolute monarchies of the preceding centuries (Hirschman 1982; Fourcade and Healy 2007).

Adam Smith argues, in *An Inquiry into the Nature and Causes of the Wealth of Nations* (1776), that humans are distinctive from other animals in the degree to which they are co-operative

Nobody ever saw a dog make a fair and deliberate exchange of one bone for another with another dog. [Smith 1776 (2012, Book 1, Chap. 2)]

Humans, on the other hand, exhibit

the propensity to truck, barter, and exchange one thing for another. [Smith 1776 (2012, Book 1, Chap. 2)]

Following the Industrial Revolution, these attitudes all but disappeared and were replaced by views that blamed a collapse of morality on the influence of commerce that was seen as commodifying human interaction; “custom is replaced by contract’. We suggest that an explanation for this change in attitude is provided by the argument presented by Adorno and Horkenheimer in *Dialectic of Enlightenment* (1944). The *Dialetic* claims that the Enlightenment led to the objectification of nature and its mathematisation, which in turn leads to ‘instrumental mindsets’ that seek to optimally achieve predetermined ends in the context of an underlying need to control external events.

Central to this process for our argument is Laplace’s treatment of probability, *Analytic Theory of Probability* (1812). Laplace can be seen as resolving the problem of uncertainty in science by building on the conception of probability introduced by Montmort and de Moivre, which were developed in to context of fixed odds games of chance rather than commerce. Laplace showed that while experimental results were ‘random’ in the sense that they were not precise, mathematics could be employed to determine the validity of the average of a set of well conducted experiments. Out of uncertainty emerges clarity. Laplace had a profound effect on all the sciences, Quetelet built social physics on his results, while Galton introduced them into the natural sciences leading to the work of Fisher and Pearson. Laplace’s conception of probability as a statistical result still dominates how the field is approached today and is the basis of much of the criticism of measure theoretic probability we have encountered.

Contemporary with Laplace, Thomas Malthus captured the anxiety of the English gentry following the French Revolution in *An Essay on the Principle of Population* that focused on scarcity. In 1836 John Stuart Mill defined political economy as being

concerned with [man] solely as a being who desires to possess wealth, and who is capable of judging of the comparative efficacy of means for obtaining that end. (Mill 1967)

Mill defended Malthus in *Principles of Political Economy* of 1848, written at a time when Europe was struck by the Cholera pandemic of 1829–1851 and the famines of 1845–1851 and Tennyson was describing nature as “red in tooth and claw”. Herbert Spencer coined the term ‘survival of the fittest’ in *Principles of Biology* (1864) after reading Darwin, who in 1871 would write

My object in this chapter is to shew that there is no fundamental difference between man and the higher mammals in their mental faculties. (Darwin 1871, p. 36)

less than a century after Smith had claimed there was a fundamental difference. Alfred Marshall synthesised Mill’s approach to economics with Darwinian metaphors of competition (Backhouse 1985, 10.1; Thomas 1991) to lay the foundations of neo-classical economics. Marshall’s 1890 definition of economics would be paired down by Lionel Robbins in 1932 as “the science which studies human behaviour as a relationship between ends and scarce means which have alternative uses”.

The simultaneous ebb away of concern for uncertainty and flow of anxiety around scarcity bring to mind Moses ben Maimon (Maimonides) who argued in *Guide for the Perplexed* (c. 1190) that God’s punishment after the Fall of Man was not so much about scarcity but uncertainty. In the Garden of Eden humans had perfect knowledge, which was lost with the Fall, and it is the loss of this knowledge which is at the root of suffering: if we know what will happen we can manage scarcity (Perlman 1997). Laplace had showed how modern science could generalise and predict in the face of uncertainty. The consequence of this was that economics focused its inquiry on competition in the face of scarcity with the emphasis being in identifying mathematical methods to optimise, essentially deterministic, expectations.

## A Pragmatic Approach to Commerce

Both Alasdair MacIntyre (2013) and Cheryl Misak (2002) are concerned with the problem that contemporary philosophy struggles to assert what is right behaviour. Misak is interested in asserting that cooperation is preferable to authoritarianism (Misak 2002, esp. Chap. 1), MacIntyre is concerned with a related problem that there is a risk that what is right comes down to what the powerful (or wilful) claim is right (MacIntyre 2013, esp. Chap. 9).

The relevance of these concerns to finance are demonstrated in the crisis related to LIBOR manipulation. LIBOR sets the benchmark interest rate used by financial institutions; if the LIBOR rate is high lenders benefit at the expense of borrowers. The consequences of manipulating the LIBOR rate upwards clearly impacts the public, holding mortgages or student loans, and will appear to benefit financial institutions. However, in many of the recent cases LIBOR was being manipulated down, reducing the cost of loans and it has been argued that there was a consequential societal benefit of manipulation in this case. One reason why LIBOR was being under-reported, other than profit generation, was to hide market concerns as to the credit-worthiness of major financial institutions and it has been claimed that this was beneficial in maintaining the financial system at a time of stress (Kaminska 2012). LIBOR manipulation is usually explained as being dubious on the grounds that it distorts trust in the market, but ‘trust’ is intangible and such arguments could succumb to more tangible, utilitarian, cases for LIBOR manipulation. We appear to lack a philosophical framework that can explain clearly why LIBOR manipulation is intolerable.

The US Department of Justice has highlighted how “LIBOR manipulation was pervasive” in some institutions and they

are concerned that too many bank employees and supervisors value coming as close to the line as possible, or even crossing the line, as being “competitive” or “aggressive”. Too many seem to be willing to take advantage of any edge—including those of dubious legality—to make money. (Cole 2013)

This highlights a problem with the current financial regulatory system. Firms are guided by a consequentialist ethic that seeks to enhance human welfare by generating profits. These activities are constrained by a deontological ethic that lays down boundaries. The US authorities can prosecute firms involved in LIBOR manipulation because LIBOR rates are instrumental in pricing derivatives, and the manipulation of the derivatives markets is illegal in the US. In the UK, LIBOR manipulation does not appear to have been illegal. This highlights an issue with the ‘carrot and stick’ regulation: the regulator must have foresight of what practices or technologies might emerge that need proscribing. For example, the growth of High Frequency Trading has been accompanied by practices that are considered dubious but outside legislation. The regulatory framework must be capable of looking into an uncertain future, something it does not appear to be at present.

In this section, having made the claim that reciprocity is a foundation of financial economics, we endeavour to robustly justify our claim and provide an explanatory hypothesis that would act as the foundation of a regulatory framework that unequivocally condemns market manipulation.

### Is it *True* that Reciprocity is a Foundation of Financial Economics?

Rubin’s discussions of emporiophobia raise an important issue discussed in the Introduction. If the claim that reciprocity is a foundation of financial economics is simply a heuristic in support of the cooperation metaphor there is no reason why economic agents *should* behave cooperatively and direct their activities in support of social cohesion. If, on the other hand, it is *true* to say that reciprocity is a key foundation of financial economics the implication is that financial economics intrinsically supports social cohesion. To appreciate this point, recall that in “The Emergence of Probability” section we argue that the genesis of mathematical probability is in Book V of *Nicomachean Ethics*, a text that addresses how an individual can live as part of a community and directly addresses the issue of social cohesion, while in “The Fundamental Theorem of Asset Pricing” section we argue that FTAP has these principles embedded within it.

Susan Haack provides us with the metaphor of knowledge as a crossword puzzle; in this sense our claim is the solution to a single clue whose validity can be assessed by comparing it to overlapping clues. We shall now consider some of these overlapping ideas in order to explore the robustness of our claim.

The FTAP is usually explained in a competitive context involving the Dutch book argument: if you priced allowing for an arbitrage a competitor would be able to act against you making a risk-less profit that would bankrupt you. The conventional explanation for the FTAP is that the objective, ‘physical process’, of hedging ensures there is no arbitrage. This argument only applies in the special circumstance of a market-maker, obliged to simultaneously give and take prices; it does not apply to market exchange in general. The problem with this heuristic is that, even for market-makers, it does not always hold. This is captured by Rama Cont and Peter Tankov when addressing pricing in markets with discontinuous prices

Unless the martingale measure is a by-product of a hedging approach, the price given by such martingale measures is not related to the cost of a hedging strategy therefore the meaning of such ‘prices’ is not clear. (Cont and Tankov 2004, 10.5.2)

Mathematically, the martingale measure exists, as Cont and Tankov had shown, but in the markets they studied the hedging argument cannot be employed. So the whole heuristic is very narrow and there is no conceptual framework supporting the prices obtained from the FTAP. In the circumstances studied by Cont and Tankov, at least, we need a better heuristic, or explanatory metaphor, than the current one. We argue that reciprocity is the basis of a better heuristic.

In fact, we reject the view that we are dealing only with a metaphor or a heuristic on the basis that the results of the Ultimatum Game. The ‘Ultimatum Game’ is an important anomaly for neo-classical economics (Thaler 1988) and is a topic of significant contemporary interest in economics, anthropology, evolutionary biology and cognitive sciences. It involves two participants and a sum of money. The first player proposes how to share the money with the second participant. The division is made only if the second participant accepts the split, if the first player’s proposal is rejected neither participant receives anything. The key result is that if the money is not split ‘fairly’ (approximately equally) then the second player rejects the offer. This contradicts the assumption that people are rational utility maximising agents, since if they were the second player would accept any positive payment. Research has shown that chimpanzees are rational maximisers while the willingness of the second player to accept an offer is dependent on age and culture. Older people from societies where exchange plays a significant role are more likely to demand a fairer split of the pot than young children or adults from isolated communities (Murnighan and Saxon 1998; Henrich et al. 2004, 2006; Jensen et al. 2007). Fair exchange appears to be learnt behaviour developed in a social context of commercial exchange, is fundamental to human society and distinguishes the sapient member of a *civitas* from the sentient animals (Henrich et al. 2004; Humphrey 1985; Fehr and Henrich 2003). Adam Smith was more accurate that Charles Darwin.

The evidence from the Ultimatum Game suggests that reciprocity, and fairness, upon which cooperation is built is an important norm beyond societies that have a relationship to Aristotelian ethics, which include Judaic, Christian and Islamic cultures. Unrecognised by Rawls, it is significant beyond liberal democracies. With this observation in mind we highlight Robert Brandom’s position, built on Wittgenstein, that there is

a pragmatist conception of norms—a notion of primitive correctnesses of performance implicit in practice that precludes and are presupposed by their explicit formulation in rules and principles. (Brandom 1994, p. 21)

Our account is that the ‘primitive correctnesses’ of the norm of reciprocity is implicit in commercial practices and was recognised by Aristotle when he investigated the nature of Justice in the context of exchange and that the norm is required to ensure social cohesion—Aristotle argued it “keeps the city together” (Broadie and Rowe 2011, 1132b34).

Despite the evidence of the Ultimatum Game, one could argue that the cross-cultural significance of reciprocity is not necessarily the correct basis for financial economics. Specifically, we have assumed that reciprocity is present because cooperation is the objective. It might turn out that this is a misguided objective, and that competition does lead to better outcomes for society. This question is of significance beyond finance and economics and we can start employing arguments in political philosophy in favour of democratic cooperation over competitive authoritarianism, such as Misak (2002). Misak argues that we can only be sure of the validity of our beliefs by putting them up for criticism and offering reasons to justify them. This is an epistemic argument that Misak claims justifies democracy: if in politics we seek the best policy we must allow our decisions to be challenged and be in a position to defend the decisions without resorting to authority; this requires that we are cooperative. Misak enables us to identify an incoherence in the thesis that the foundation of financial economics is *competition*: the argument is created out of a cooperative principle (in science) but concludes that a competitive ethic is preferable (in finance).

Misak’s argument creates a link between political philosophy and epistemology: it connects democracies to science (Misak 2002, p. 94). The argument we develop in “The Emergence of Probability” section comes out of Borkenau’s *The Transition from the Feudal to the Bourgeois World View* (1934) that argues modern science emerges out of the capitalist system: a connection between science and commerce. Marion Fourcade and Kieran Healy point out that *doux-commerce* argues that “[c]ommerce teaches ethics mainly through its communicative dimension, that is, by promoting conversations among equals and exchange between strangers.” (Fourcade and Healy 2007, p. 287): a connection between commerce and democracies. We appear to have a triad consisting of markets, science and democracy connected by the requirement to test the validity of our ideas by putting them up for criticism and then defending them.

### An Explanatory Hypothesis

In the triad, markets are anterior to both science and democracy. We offer an explanation for this observation: markets are centres of communicative action. Moreover, because markets are fast-moving, time is compressed and practices evolve rapidly, metaphorically they are unconscious social research laboratories where the practices of communicative action are refined.

The term ‘communicative action’ was coined by Jürgen Habermas as he sought to develop a more optimistic assessment of the Enlightenment than that presented by Adorno and Horkeheimer in *The Dialectic of the Enlightenment*. In *Structural Transformation of the Public Sphere*, Habermas argues that during the seventeenth and eighteenth centuries public spaces emerged, the public sphere, which facilitated rational discussion that sought the truth in support of the public good. In the nineteenth century, mass circulation mechanisms came to dominate the public sphere and these were controlled by private interests. As a consequence, the public became consumers of news and information rather than creators of a consensus through engagement with information. Having undertaken this analysis of the contemporary state of affairs, Habermas sought to describe how the ideal of the Enlightenment public sphere could be enacted in the more complex contemporary (pre-internet) society and his *Theory of Communicative Action* is the result.

Central to *Communicative Action* is a rejection of the dominant philosophical paradigm, the ‘philosophy of consciousness’, that is rooted in Cartesian dualism; the separation of mind-body, subject-object, concepts and is characterised by Foundationalism; philosophy is required in order to demonstrate the validity of science and the validity of science is based on empiricism, and certain views specific to the social sciences; such as that society is based on individuals (atoms) interacting, so that society is posterior to individuals and that society (a material, extending the physical metaphor) can be studied as a unitary whole, not as an aggregate of individuals.

This dominant paradigm sees language as being made up of statements that are either true or false and complex statements are valid if they can be deduced from true primitive statements. This approach is exemplified in the standard mathematical technique of axiom-theorem-proof. Habermas replaces this paradigm with one that rests on a Pragmatic theory of meaning that shifts the focus from what language says (true or false) to what it does. Specifically, Habermas sees the function of language as being to enable different people to come to a shared understanding and achieve a consensus, this is defined as discourse. Because discourse is based on making a claim, the claim being challenged and then justified, discourse needs to be governed by rules, or norms. The most basic rules are logical and semantic, on top of these are norms governing procedure, such as sincerity and accountability, and finally there are norms to ensure that discourse is not subject to coercion or skewed by inequality.

There is an Asian description of a market as “Two women and a duck” and the essence of the proverb is that if two women, who are characterised as talkative, and a duck come together, eventually the value of the duck will be determined—knowledge is created. More generally, the market mechanism requires that two agents agree the price of an asset. If the price is determined by a single unit we have a non-market mechanism involving a private monopoly or state intervention. These examples in the context of the preceding discussion justify our claim that markets are centres of communicative action that aim to achieve consensus on the price of assets.

This claim has some technical implications. Firstly, market discourse is a specific type of discourse that would depend on particular norms of discourse. We propose that reciprocity is a key norm of market discourse that is required to ensure impartiality, as Aristotle observes (Broadie and Rowe 2011, p. 35). Another norm that appears to have been important in market discourse is ‘charity’. This seems peculiar in the contemporary setting and the common interpretation of charity as altruistic giving, which is at odds with reciprocity, rather than the traditional definition as care for others (‘benevolence’ would be a better contemporary alternative). However, it is worth noting that one reading of Shakespeare’s *The Merchant of Venice* is as of a study of the four natures of classical love, with Antonio, ‘the merchant of Venice’ characterising charity (*caritas*/*agape*). More practically, British finance rests on foundations laid by Quaker institutions, reflected in the names of Barclays, Lloyds, Waterhouse, and Coopers. The financial success of these Quaker families was built on a reputation for honesty and sincerity, a strong social network built on democratic principles and a tradition for open discourse (Walvin 1998). Above all, a Quaker banker needed to conform to religions precepts that included charity and is captured in the proverb

“Well, Friend”, said the Quaker Banker, “Tell me the answers to these questions so that I may help you in your projects, for you have opportunities: Firstly, how much do you seek to borrow? For how long? And *how will you repay the loan plus its interest*?” These are the issues all good bankers must explore. (my emphasis)

The second implication concerns the role of mathematics in finance. Mathematics is widely regarded as delivering indubitable results, and on this basis a price derived mathematically has authority. However, in the context of communicative action, language is not a truth carrier but rather it is a linguistic device to enable the transmission of understanding. This is the role of Fibonacci’s mathematics in medieval finance, highlighted by Sigler (Fibonacci and Sigler 2003, Introduction); it enabled a calculation to be written down that could then be copied, modified and improved by others. In not recognising this role for mathematics, financial economics has been perceived by many as being capable of delivering accurate asset prices in the face of radical uncertainty. The Black–Scholes pricing formula was once claimed to be the most successful equation in economics (Ross 1987, p. 332).

However since Black Monday in 1987 market practitioners have been more sceptical of the accuracy of the Black–Scholes equation, and as Haug and Taleb (2011) point out, traders rarely use the formula and prefer practical heuristics. In contemporary markets, the prices of standard, exchange traded derivatives are determined by ‘the market’, while exotic, over-the-counter derivatives are too complex to be priced analytically. Donald MacKenzie observes that, in practice, financial economics provides “a benchmark ‘fair’ price that facilitates *negotiation*” (MacKenzie 2008, p. 257, my emphasis). Problems that MacKenzie has investigated: the super-portfolio in relation to the failure of Long Term Capital Management (MacKenzie 2003b); and the choice of 0.3 as the correlation parameter used in pricing CDOs in the lead up to the Credit Crisis (MacKenzie 2011) are both problems of a monism related to a belief in the indubitability of mathematics. Replacing the negotiation between market agents with an algorithm that delivers a theoretical price replaces ‘knowledge’, generated through communication, with dogma. This is an almost trivial observation to (successful) market participants (e.g. Tett 2009; Beunza and Stark 2012; Duhon 2012, especially Chap. 12).

Gabrielle and Reuven Brenner identify ‘speculators’ as market participants that bet on a miscalculation of the odds quoted by the market and the reason why speculators are regarded as socially questionable is that they have opinions that are explicitly at odds with the consensus (Brenner and Brenner 1990, p. 91; Beunza and Stark 2012, p. 394). A good description of the process that speculators are involved in is given by Beunza and Stark (2012), which clearly explains how mathematics is just one method of testing the validity of a trader’s intuition.

Rather than seeing traders as seeking a profit in a competitive arena, we can see traders as seeking the truth in the face of market uncertainty; in offering a price they are making a claim. William James recognised this association when he uses a financial metaphor to explain *Pragmatism’s Conception of Truth*

Truth lives, in fact, for the most part on a credit system. Our thoughts and beliefs ‘pass,’ so long as nothing challenges them, just as bank-notes pass so long as nobody refuses them. But this all points to direct face-to-face verifications somewhere, without which the fabric of truth collapses like a financial system with no cash-basis whatever. You accept my verification of one thing, I yours of another. We trade on each other’s truth. But beliefs verified concretely by SOMEBODY are the posts of the whole superstructure. (James 2010, p. 80)

Arjun Appadurai offers another perspective on the behaviour of traders in the face of market uncertainty with the observation that speculators

believe in their capacity to channel the workings of chance to win in the games dominated by cultures of control …[they] are not those who wish to “tame chance” but those who wish to use chance to animate the otherwise deterministic play of risk [quantifiable uncertainty]”. (Appadurai 2011, pp. 533–534)

Appadurai was motivated to study finance by Marcel Mauss’ essay *Le Don* (‘The Gift’), exploring the moral force behind reciprocity in primitive and archaic societies and goes on to say that the contemporary financial speculator is “betting on the obligation of return” (Appadurai 2011, p. 535), and this is the fundamental axiom of contemporary finance. David Graeber also recognises the fundamental position reciprocity has in finance (Graeber 2011), but where as Appadurai recognises the importance of reciprocity in the presence of uncertainty, Graeber essentially ignores uncertainty in his analysis that ends with the conclusion that “we don’t ‘all’ have to pay our debts” (Graeber 2011, p. 391). In advocating that reciprocity need not be honoured, Graeber is not just challenging contemporary capitalism but also the foundations of the *civitas*, based on equality and reciprocity (Graafland 2010, p. 235).

The argument that we have presented is that the norm of reciprocity is implicit in the practice of commerce because it enables participants in a market to converge at a consensus of the price of an asset: it is a rule of market discourse. Reciprocity becomes explicit in Aristotelian ethics and then in the early conceptions of mathematical probability. The norm becomes obscured as a consequence of the ‘rationalisation’ process that followed the Cartesian revolution, that aimed to remove doubt from philosophy (Bernstein 2013, Chap. 1), led to Hume’s introduction of the fact/value dichotomy (Wilber and Hoksbergen 1986) and the Laplacian revolution, that appeared to resolve the issue of uncertainty in science. In the process, theory and practice, subject and object, facts and values, means and ends are all separated. In this environment *ex cathedra* norms, in particular utility (profit) maximisation in the face of scarcity, encroach on commercial practice. This is exemplified by the 1950 English court case Buttle v. Sunders ([1950] 2 All ER 193) where it was judged that ‘my word is my bond’ was subordinate to the profit maximisation principle. With the Nixon Shock and collapse of the Bretton Woods system of fixed exchange rates uncertainty re-emerged in the markets resulting in the failures of a financial economics that assumes an ergodic economy and the existence of objective functions. Regarding markets as centres of communicative action is essentially a response to acknowledging that markets are unpredictable but exchange needs to take place in this indeterministic environment.

### Policy Implications of the Explanatory Hypothesis

Pragmatism demands that “we identify the meaning of an idea with its sensible effects” (Bacon 2012, p. 27). This leads us to the question of how would our experience of finance changes if we accepted that reciprocity is the basis of financial economics because markets are centres of communicative action.

Caplan (2007) considers markets in the context of profit seeking, consequentially efficient, mechanisms for distributing resources. We argue that markets are centres of communicative action built on a norm of reciprocity that stipulates that a profit is only possible if accompanied by risk. There is widespread public dissatisfaction with Caplan’s view and concern that profit seeking financial agents were responsible for the Financial Crisis of 2007–2009 (e.g. Caccioli et al. 2009 which is influential on the widely cited Haldane and May 2011; Simsek 2013). The pursuit of profit in the face of an uncertain future makes the financial system less stable, and so less effective at distributing resources.

Since the Credit Crisis there has been an explosion of alternative financial mechanisms such as peer-to-peer lending and ‘crowdfunding’ that relate to long-established not-for-profit institutions such as Friendly Societies and Credit Unions. There is a view that the growth of these mechanisms is a result of conventional finance not effectively funding entrepreneurs (Collins et al. 2013, p. 12). The common feature of these new mechanisms is that, by employing new digital technologies, they enable the financing of projects directly by individuals, without the intermediation of financial institutions. Because intermediaries; banks, asset manages or insurers, have a fiduciary duty that is interpreted as maximising the returns to their investors/depositors, they often fail to fund long term projects that do not offer an immediate return. By enabling direct investment, not focused on short term profits, the emerging financial mechanisms are frequently associated with the funding of projects that sustain communities over the long term. If the claim that reciprocity is a key foundation of financial economics is believed, then the criteria for assessing an investment is whether there will be a reciprocal exchange, not if it maximises the returns to the investor. As well as justifying the financial basis of these emergent, not-for-profit, financial mechanisms this approach would accommodate the concept of intergenerational reciprocity as advocated by Stern (2008), discussed in the Introduction.

The new financial mechanisms are currently being reviewed by regulators: UK Financial Conduct Authority (FCA) Consultation Paper 13/13 and US SEC RIN 3235-AL37. There is concern that regulators will force the emergent mechanisms to mimic existing structures, which are profit maximising, destroying their beneficial distinctiveness and the FCA consultation was debated in the UK Parliament (18 December 2013, ‘Crowdfunding and the FCA’). The reasonable concern of the regulators is that naïve investors will be tempted into schemes without merit by manipulative financiers and the role of the regulator is to ensure ‘fairness’ between competing investment opportunities. Our response to this is that the modern financial system alienates investors from their investments. As explained in the *Dialectic of the Enlightenment* investors have become passive consumers of financial products and do not actively engage in finance. The new, internet based technologies, provide the possibility of creating genuine public spheres in which investors can engage directly with markets as centres of communicative action. Policy towards these emergent financial mechanisms should not be guided solely on their ability to maximise profits, but also their effectiveness in funding economic activity and their ability to promote investor engagement in finance. The evidence in this paper suggests that the opportunities outweigh the risks of the emergent mechanisms.

A recent example of the process of investors’ alienation emerged in 2013 as it became apparent that the English Episcopal Church (the Church of England) was simultaneously campaigning against, so called, pay-day loan companies, who offer small, short term loans at high interest rates, while investing in them. It could be argued that these loans do not contravene the norm of reciprocity if the interest rate charged creates an equality between what the lenders give and what they expect to be repaid. If the interest is a pure credit risk premium they are not being usurious, ‘asking for more than what was given’. However, if the lenders do not really expect to be repaid they do not adhere to the Quaker’s injunction that a banker, in order to be charitable, must be confident in the borrower’s ability to re-pay the debt.

Jonathan Levy describes how the alienation of investors from their investment was important in the emergence of Mortgage Backed Securities in the US in the 1880s and how this made it difficult for the investor to be charitable towards the borrower (Levy 2012, pp. 162–165). The phenomenon was repeated in the lead up to the Credit Crisis. Banks were happy to offer loans to people who had no real prospect of repayment, simply because they were profitable, resulting in an explosion of sub-prime lending that was at the heart of the Crisis.

Levy also describes how in the 1870s fraternal (mutual) life insurance mechanisms were attacked by capitalist life insurance companies employing the rhetoric of Laplace and claiming that there was certainty in the actuarial science (mathematics) that the life firms employed (Levy 2012, p. 198). The life firms seemed oblivious to the fact that the mutuals had emerged partly in response to the failure of actuarially managed firms which had collapsed in the aftermath of the 1873 panic. This attitude of the nineteenth century corporate insurers is particularly interesting in the context of our argument given that the origins of actuarial science are in an explicitly charitable culture. The first mathematically managed pension fund was the Scottish Ministers’ Widows Fund, established in 1744. The Presbyterian Church of Scotland recognised its obligations to the widow’s of its ministers and two Edinburgh ministers, Robert Wallace and Alexander Webster, acted. Webster gathered statistical data while Wallace reviewed the emergent literature and in 1743 Wallace was able to calculate premiums that would deliver defined widows’ benefits with a precision that resulted in a fund whose modelled value never deviated more than 5% from the realised over the next thirty years (Dunop 1992; Bremner 1992; Hare 1992). Wallace and Webster can be seen as synthesising the three ‘Christian virtues’ to create actuarial science: charity, for the widows, faith in Webster’s statistics and hope, in Wallace’s use of probability.

In the contemporary context, there is significant concern that the involvement of mathematics in finance is not so positive. In their submission to the Parliamentary Commission on Banking Standards the Bank of England was highly critical of how some firms have recently used advanced mathematical techniques to ‘pull the wool’ over the eyes of the regulator (PCBS 2013, para. 89, v. II). The issue here is one of sincerity, identified by Habermas as being a critical norm in communicative action. Yuthas et al. (2002) describe the role that corporate annual reports play in ensuring sincerity in commercial practice, we, and the regulator, are concerned here how certain technologies enable insincerity. For example, the practice of quote, or order, ‘stuffing’ in high frequency trading, issuing large numbers of orders to an exchange and then cancelling them within a tenth, often a hundredth, of a second is widely regarded as being an attempt to manipulate the market. While acknowledging this concern, the UK Government Office for Science has not advised that any legislation should be enacted in order to prevent the practice. They use a sporting metaphor to explain that there is a competitive market in exchanges, and legislation would discourage trading on the UK exchanges (Foresight 2012, Sect. 8.2). This position contrasts with the German Parliament that has legislated on the issue (Hochfrequenzhandelsgesetz, 28/2/2013). If the markets were not regarded as competitive arenas but were seen as centres of communicative action order stuffing would not be tolerated as it contravened the norm of sincerity/truthfulness.

The current regulatory framework can be characterised as balancing a ‘consequentialist’ ethic: profit seeking in order to maximise social welfare; with a ‘deontological’ ethic: that defines rules, such as capital reserving, designed to constrain the profit seekers. Given recent financial scandals, such as LIBOR manipulation and the ‘London whale’ (Permanent Subcommittee on Investigations 2013) where advanced mathematics were employed to significantly reduced the reported risks, this ‘carrot and stick’ approach seems flawed. An implication of reorienting financial economics to focus on the markets as centres of ‘communicative action’ is that markets could become self-regulating, in the same way that the legal or medical spheres are self-regulated through professions. This is not a libertarian argument based on freeing the consequential ethic from a deontological brake. Rather it argues that being a market participant entails restricting norms on the trader, such as reciprocity, sincerity and charity, that support knowledge creation, of asset prices, within a broader objective of social cohesion. Within this framework market manipulation, through order stuffing, gaming the regulations or forging LIBOR quotes, would be clearly illicit and punishable by exclusion from the profession.

The Bank of England’s views (PCBS 2013, para. 89, v. II) are related to concerns identified by professional bodies who have been working on responding to the Parliamentary Commission’s recommendations. While professional bodies are positive about engaging retail and commercial bankers with the ethics agenda, they have found it more challenging to engage ‘quantitative finance’ with ethics (Brogan 2013), reflecting the case made in West (2012). An explanation for this could be in the fact that most professionals working in quantitative finance are coming out of academic fields, such as mathematics and physics, where there is little or no focus on ethical issues. This paper can assist professional bodies in bringing the ethics agenda into quantitative finance.

## Conclusion

The genesis of this paper was in the recognition of a formal equivalence between the Cox–Ross–Rubinstein binomial model for pricing derivatives (1979) and the canonical origin of mathematical probability in the Pascal and Fermat solution to the Problem of Points (1654). The structural similarity is obvious and immediately raises the question of how probability was conceived in the seventeenth century. This question is informed by the fact that the probabilities in the Cox–Ross–Rubinstein model are, today, understood in terms of Kolmogorov’s measure theoretic probability, and not in terms of objective (frequentist) or subjective (Bayesian) probability. Exploring the scholarship, notably Sylla, Bellhouse, Franklin Kaye and Hadden, we understand that, before Montmort and de Moivre, probability was based on Aristotelian ethics and the requirement to maintain equality in exchange—reciprocity—in order to promote social cohesion. In effect, mathematical probability originates in a synthesis of Fibonacci’s commercial mathematics and Scholastic analysis of exchange.

The Cox–Ross–Rubinstein model is today understood in the context of the Fundamental Theorem of Asset Pricing (FTAP), which states that a necessary and sufficient condition for a market to preclude arbitrage is the existence of a martingale measure. We associate no-arbitrage with fairness—equality in exchange—and martingale measures with seventeenth century conceptions of probability to claim that the FTAP is simply a re-stating of seventeenth century ideas and so reciprocity is a key component of contemporary financial economics. This is significant in the context of Granovetter’s discussion of embeddedness in economics (Granovetter 1985). It is conventional to assume that mainstream economic theory is ‘undersocialised’: agents are rational calculators seeking to maximise an objective function and this is implicit in Horrigan’s and Frankfurter and McGoun’s criticism of modern financial economics. The argument presented here is that a central theorem in contemporary economics, the FTAP, is deeply embedded in social norms, despite being presented as an undersocialised mathematical object. The consequence of this result is that we can retain the paradigm of ‘New Finance’ while working to ensure that it is a ‘nice place ethically’.

The critical difference between this paper and the work of Horrigan and Frankfurter and McGoun is that we identify a moral dimension to probability theory. We are able to do this by considering the ‘pre-history’ of mathematical probability and the question arises, why was the moral dimension lost? We offer an explanation for the disappearance of the link in the context of Adorno and Horkenheimer’s thesis presented in *Dialectic of the Enlightenment*. We highlight how in the nineteenth century science replaces uncertainty with Laplacian determinism (Gigerenzer 1989; Hacking 1990) and scarcity comes to dominate economics. It was within this framework of a strict fact/value dichotomy that the Black–Scholes and Cox–Ross–Rubinstein models were developed. We justify abandoning the fact/value dichotomy on the basis of Pragmatic philosophy, which challenges the philosophical framework laid in the Cartesian revolution and creates links to modern Virtue Ethics.

We justify the validity our claim by employing a Pragmatic approach and look at ‘the conceivable effects of a practical kind’ that our claim has. We start by highlighting that the conventional heuristic supporting the FTAP is inadequate (Cont and Tankov 2004, 10.5.2) and that reciprocity offers a stronger explanation. We then employ the results of the Ultimatum Game to offer our main justification for the claim that reciprocity is at the heart of financial economics. The Ultimatum game is studied from the context of both social anthropology and cognitive sciences and the argument we make, based on social phenomena, in favour of ‘moral markets’ is distinctive from approaches grounded in the cognitive sciences, such as Zak and Jensen (2010). The essential difference is that we reject what Habermas characterised as the ‘philosophy of consciousness’ and we consider how human behaviour is determined by social practices, rather than how social practices are a consequence of neurological phenomena. One possible explanation for for results of the Ultimatum Game is that reciprocity is optimal in the face of uncertainty (Delton et al. 2011), this connects to our conjecture that the decline in concern for uncertainty led to a decline in emphasis of reciprocity. We suggest a connection with Brandom’s thoughts on norms being implicit in practice and become explicit in theory, with the norm of reciprocity being important in dealing with uncertainty.

Having used the empirical results of the Ultimatum Game to support our claim we are still exposed to the question as to whether reciprocity, and the coagmentative concept of cooperation, are optimal for society. Our final reason for justifying the central claim is adapted from Misak’s justification for cooperation in politics and we highlight connections between commerce, democracy and science. On this basis we argue that markets should be regarded as centres of ‘communicative action’ governed by rules of market discourse identified here as reciprocity, sincerity and charity. The argument that communicative action is important in understanding commerce is not novel (Yuthas et al. 2002), the contribution of this paper is in identifying reciprocity as a norm of discourse in the context of communicative action. The coherence of this claim is based on the fact that Habermas’ theory of communicative action was developed in response to Adorno and Horkenheimer’s *Dialectic of the Enlightenment*. In this scheme mathematics is a linguistic device to enable consensus formation in the face of radical uncertainty, not as the indubitable determinant of a true price and we briefly discuss this observation in the context of some contemporary scholarship on markets.

Our main claim is that reciprocity is a key foundation for financial economics, the working hypothesis to explain the claim is that markets are centres of communicative action. Our paper ends with a discussion of how our claim and explanatory hypothesis would affect our experience of markets. Specifically we argue that not-for-profit mechanisms should be encouraged while certain activities, such as order stuffing, should be inhibited and market manipulation, in general, cannot be tolerated on the basis that it is insincere. More generally we call for greater public engagement with financial practice and the substitution of ‘carrot and stick’ by ‘professional’ regulation of financial practice. The objective is that this paper can contribute to this process, not least by contributing to a reversal in the trend for economics education to promote greed, as identified in Wang et al. (2011) and supports the arguments in Jackson (2010) and West (2012).

We note that Beckert (2009) has recently pointed out that the coordination necessary for markets to function, involving ‘valuation’, ‘cooperation’ and ‘competition’, rests on participants as having “stable reciprocal expectations”, necessary because of the extreme (aleatory) uncertainty that is a feature of markets. This paper is aligned to Beckert in acknowledging the centrality of these concepts, but we have synthesised the valuation and cooperation problem while Beckert suggests they are analysed separately.

We see this paper as having the potential to motivate further research in a number of fields. Obviously there is the potential to re-interpret the whole canon of financial economics on the basis of communicative action. While our aims are similar to those of Horrigan and Frankfurter and McGoun, our approach does not entail the replacement of financial economic theory, just its re-interpretation in a manner advocated by Rubin.

We highlight the triad of markets, politics and science and observe that in the sixteenth and seventeenth century this triad was characterised by a number of significant figures: Gresham, Stevin, Huygens and Newton. Despite the significance of all these individuals in the emergence of modern science the impact of interactions between markets, politics and science at this time has not featured in the historical literature. This could be an interesting topic for further research.

Poincaré argued that the value of mathematics was in being able to perform experiments when physical experimentation is not possible—the mathematical identification of the Higgs Boson decades before it was technically feasible to identify it empirically is a case in point. This paper raises a question: how do we know that cooperation built on reciprocity does lead to better outcomes for society in the face of uncertainty? This cannot be shown through experiment but it could be investigated mathematically. For example, recent models analysing whether the proliferation of financial instruments leads to instability (e.g. Caccioli et al. 2009—which is influential on the widely cited Haldane and May 2011; Simsek 2013) are based on the assumption that agents are seeking to maximise utility; Sahlins’ ‘negative reciprocity’, rather than acting in a framework of ‘balanced reciprocity’. An interesting research question would be to use the techniques of complex network theory to investigate what types of financial networks emerge, and are maintained, on the basis of either ‘negative’ reciprocity, allowing for default in the context of the ‘Prisoner’s Dilemma’ game, or ‘balanced’ reciprocity, and then analyse if different network topologies are more, or less, effective in financing or resilient to financial shocks. This could provide some insight into the role balanced reciprocity plays in supporting markets, even large markets such as the LIBOR and foreign exchange markets that do not match the scale of Sahlins’ ‘tribal sector’, potentially explaining the embeddedness of reciprocity in financial economics.
